# Isolated Nocturnal Hypertension in Children

**DOI:** 10.3389/fped.2022.823414

**Published:** 2022-02-18

**Authors:** Midori Awazu

**Affiliations:** Department of Pediatrics, Tokyo Metropolitan Ohtsuka Hospital, Tokyo, Japan

**Keywords:** isolated nocturnal hypertension, children, target organ damage, masked hypertension, clinic blood pressure, masked isolated nocturnal hypertension

## Abstract

Isolated nocturnal hypertension (INH) is attracting attention because it has been shown to correlate with target organ damage as well as cardiovascular events in adults. INH has also been reported in children especially in those with underlying diseases including chronic kidney disease and some studies reported association with markers of early target organ damage. INH occupies the majority of nocturnal hypertension. On the other hand, masked hypertension is largely attributed to INH. INH is usually diagnosed by ambulatory blood pressure monitoring. Recently, it became possible to monitor sleep blood pressure by an automated home blood pressure device feasible also in children. The epidemiology, methodology and reproducibility, pathophysiology, relation to target organ damage, and treatment of INH in children will be reviewed here along with adult data.

## Introduction

The condition isolated nocturnal hypertension (INH) was highlighted in 2007 by Li et al. (1) although the term had been used since late 1990's. It is defined as hypertension during the night with normal daytime blood pressure (BP) when assessed by ambulatory BP monitoring (ABPM). Specifically, INH is defined in adults as nocturnal BP over 120 and/or 70 mmHg in the presence of daytime BP <135/85 mmHg (2). American guidelines recommend more restrictive values as nocturnal BP ≥ 110 and/or ≥ 65 mmHg (3).

**Figure 1:**
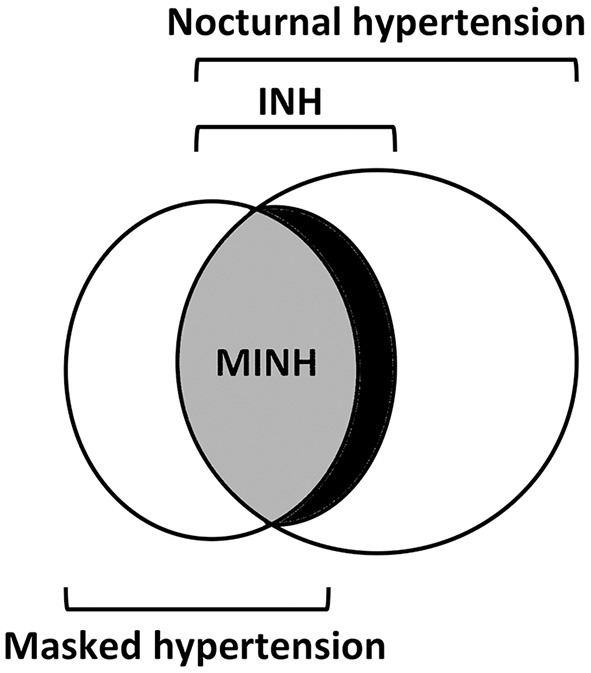
The overlap between subjects with nocturnal hypertension, isolated nocturnal hypertension, masked isolated nocturnal hypertension, and masked hypertension. More than one-third of the subjects with nocturnal hypertension had isolated nocturnal hypertension **(INH)**. INH consists largely of masked INH **(MINH)**. Masked hypertension, on the other hand, is largely attributed to INH.

Nocturnal BP has been reported to better predict target organ damage (TOD) and cardiovascular events in adults compared with daytime BP or 24-h mean BP. It can occur both in dippers and non-dippers but is considered superior to non-dipping status as a marker of cardiovascular disease risk (4). Because of this value of nocturnal BP, the European Society of Hypertension has incorporated nocturnal BP into the definition of white coat hypertension and masked hypertension (2). White coat hypertension is defined by an elevated clinic BP with normal ambulatory or home BP. Masked hypertension is a condition characterized by a normal clinic BP with an elevated BP out of the clinic. Both could be identified by the ABPM of home BP monitoring (HBPM), and INH is another such phenotype. When INH is accompanied by normal clinic BP values, INH is designated as masked INH (MINH) (Figure 1).

## Epidemiology

### Adults

The prevalence of INH in adults varies depending on the study subjects and the criteria. The prevalence are 10.9, 10.5, 10.2, 7.9, 6.0, and 19% in a population-based sample of Chinese, South Africans, Japanese, Eastern and Western Europeans, and African Americans, respectively (5). INH seemed to be more common in Asians and Africans than Europeans. In the Pressioni Arteriose Monitorate E Loro Associazioni (PAMELA) study, which enrolled 2021 participants in Italy, however, INH was observed in 11.4% of the participants (6). In the Jackson Heart Study, INH was present in 19% of the entire cohort (7). The study by Salazar et al. (8) conducted in Argentina reported the prevalence of INH to be 12.9%. Its prevalence was similar among non-hypertensive clinic BP states (optimal, normal, and high-normal BP) (8). In a recent study from Korea, the prevalence of INH in the general population was very high (22.8%) (9). Furthermore, in contrast to Salazar et al. (8) study, the prevalence of INH was highest in subjects with clinic prehypertension. Thus, prehypertensive subjects are at risk for INH and ABPM may be warranted.

In Li et al. (1) study of a Chinese cohort, which included subjects over 12 years, individuals with INH were older, reported more alcohol intake, and had faster nocturnal pulse rate, and higher serum total and LDL cholesterol and blood glucose levels compared to those with ambulatory normotension. In the PAMELA study, INH subjects had many characteristics of the metabolic syndrome, and were older and heavier than the normotensives (6). Multivariate analyses showed that the independent correlates of INH were age, waist, and neck circumferences (8). Individuals with INH also had higher prevalence of type 2 diabetes mellitus compared with normal BP controls (19 vs. 10%) (7).

The prevalence of INH was reported to be high in patients with chronic kidney disease (CKD) (20.4%) (10). There were association between INH and age, estimated glomerular filtration rate, and clinic diastolic BP. Diseases associated with INH other than CKD include diabetes (11). Sleep apnea causes nocturnal BP increase, but there has been no study examining its association to INH.

INH consists largely of MINH (Figure 1). In Li et al. (1) study, the subjects with INH who had elevated clinic BP were only 5.4%. In Salazar et al. (8) study, the prevalence of INH was lower in patients with clinic hypertension than in normotensive patients (7.4 vs. 17.2%). From the other way around, masked hypertension is largely attributed to INH. Data from the Spanish ABPM Registry in treated hypertensive subjects showed that masked hypertension was very common mostly because of MINH (12).

The prevalence of MINH with type 2 diabetes was reported to be 7.2 to 30% in adults (11, 13). In renal transplant recipients, the prevalence was 19% (14). Among African American adults with antihypertensive medication, the prevalence of MINH was high in those with diabetes compared with those without (48.8 vs. 33.2%) (15). This implies that renal transplant, diabetes, and/or antihypertensive medication are a risk for MINH.

Masked hypertension has the long-term risk of developing sustained hypertension, which is also thought to apply to MINH (16).

### Children

We investigated the frequency and clinical characteristics of INH in 198 Japanese children and young patients seen in our pediatric nephrology clinic. INH was detected in 32 patients (16%) (17). The frequency of INH in subjects with no underlying disease (primary hypertension) was 35.6%, 18.2% in those with renal disease, 26.3% with endocrine disease, and 11.1% with cardiac disease (17). In a recent Seeman et al. (18) study in children with untreated ambulatory hypertension, INH was seen in 19%. The etiology of INH was primary in 21% and secondary in 79%, renoparenchymal in 74%, steroid-induced in 5%, and diabetes mellitus in 5% of INH patients. The frequency of INH in 456 children with CKD was shown to be 13.4% from the Cardiovascular Comorbidity in Children with Chronic Kidney Disease Study (19). In renal recipients, the frequency of INH varied from 16 to 43% (20). Prednisolone dose was associated with nocturnal diastolic pressure and steroid withdrawal restored the nocturnal dip (21, 22). Other specific diseases in which INH has been reported in children include type 1 diabetes, obesity, a history of preterm birth, multicystic dysplastic kidney, and autosomal dominant polycystic kidney disease (23–28). The frequency of INH in normal children is unknown.

The frequency of MINH in Japanese children seen in pediatric nephrology clinic was 8.1% (17). MINH consisted of primary hypertension (56.3%), renal disease (37.5%), and endocrine disease (6.3%) (17).

In a long-term follow-up study enrolling 272 healthy clinic normotensive youths (6–18 years), development of sustained hypertension was 7% in masked hypertension compared to 0.6% in normotensive youth (29). Thus, also in children, masked hypertension, which includes MINH, is a precursor of sustained hypertension.

## Methodology and Reproducibility

### Methodology

ABPM is the standard method to measure out-of-office BP levels, especially for nocturnal BP measurement. However, its use is limited because of its cumbersome nature, patient's low tolerability, unavailability of devices, and low repeatability. HBPM can solve these limitations of ABPM. Automated HBPM devices have been developed for the measurement of nocturnal BP (30). Three BP measurements usually three times at 1-h intervals are available. A recent meta-analysis reported that the significance of nocturnal BP measured by HBPM was similar to that measured by ABPM (31). The advantages of HBPM for measuring out-of-office BP levels are higher reproducibility (see below), reliability, practicality, and its lower costs compared with ABPM. Thus, HBPM could be a complementary or even an alternative technique to ABPM for the diagnosis of nocturnal hypertension. There is, however, no consensus on the optimal protocols for nocturnal HBPM at present.

A previous study showed that cuff inflation increased arousal and wakefulness in subjects who underwent polysomnography and ABPM at the same time (32). Thus, elevated nocturnal BP could be due to arousal response. Recently developed cuff-based watch-like wrist devices are less intrusive and may prove to be useful (33). Non-invasive continuous BP monitoring technologies, although largely limited to the research setting, may become an alternative to cuff-based ABPM (34).

### Reproducibility

The reproducibility of INH defined by ABPM has been reported to be poor on both long-term and short-term (1, 35). In addition, isolated daytime hypertension was also poorly reproducible (35). The study concluded that a single 24-h ABPM was not able to be used to identify individuals with INH. There were limitations in the study, however, such as small sample size, characteristics of the study participants, and racial difference. The results from Cuspidi et al. (6) study showed that reproducibility was 72.5% in the period of 4 weeks.

Reproducibility of HBPM, on the other hand, in measuring nocturnal BP levels was reported to be high (36). The study, however, used the nocturnal BP cutoff threshold used in ABPM. It is not known whether nocturnal BP measured by HBPM over 2 weeks is equivalent to that measured by ABPM over 1 night.

In children and adolescents, it has recently been shown that 7-day home BP (HBP) had similar reproducibility as 24-h ambulatory BP (ABP) (37). The agreement between nocturnal HBP and ABP in identifying individuals with INH (defined as ≥ 95th percentile for nocturnal ABP) was 82%.

## Pathophysiology

Li et al. (5) proposed that INH occurs due to blunted sodium excretion and high sodium intake. Chinese and Japanese who have high frequency of INH consume high sodium diets. Furthermore, urinary sodium excretion was shown to be lower in patients with INH (1). Arterial stiffness could be another cause of INH. Patients with INH had similar increased arterial stiffness and wave reflections as sustained hypertensives despite lower 24-h BP (1). In support of this hypothesis, aortic pulse wave velocity was the only independent differentiator between INH and ambulatory normotension in the Swedish study (13). Arterial stiffness was shown to be more closely related to nocturnal than daytime BP (38). There are also other potential mechanisms including increase in sympathetic nervous system and renin–angiotensin–aldosterone system activity, renal dysfunction, insulin resistance, obstructive sleep apnea, obesity, aging, stress, and diabetes (39). Some of the mechanisms may provide a basis for therapeutic interventions.

## Target Organ Damage

### Adults

Nocturnal BP in general is known to be superior to daytime BP or 24-h mean BP as a predictor of TOD in adults (11, 40). INH defined by ABPM has also been linked to TOD and cardiovascular events (11). With respect to subclinical cardiovascular TOD, participants with INH defined by ABPM showed increased arterial stiffness and greater left ventricular mass compared with those of a controlled BP group (1, 7). A systematic review revealed, however, that subclinical TOD including ventricular hypertrophy, arterial stiffness, and proteinuria was not always associated with INH (41). In African Americans, on the other hand, INH was associated with higher risks of developing CKD as sustained hypertension (42). In Chinese CKD patients, multivariable regression analyses showed that INH was associated with an increased risk for renal events and cardiovascular events compared with nocturnal normotension, even when adjusted for clinic BP, 24-h BP, or daytime BP (10). Further studies have to be performed to determine the prognostic value of INH over multiple ethnic groups.

In patients with type 2 diabetes, MINH defined by ABPM was associated with increased arterial stiffness and higher central BP (13).

Subjects with MINH defined by HBPM are also at high risk of future CVD events (43). In Japanese with a history of CVD or at risk for CVD, MINH defined by HBPM was associated with (1) greater cardiovascular TOD as represented by a higher urine albumin and an increased risk for total CVD events (36). The magnitude of total CVD event risk in the MINH group was intermediate between those with controlled BP and those with sustained hypertension.

### Children

Lee et al. (44) reported that carotid intima-media thickness (CIMT) was higher in diabetic children and adolescents with nocturnal hypertension. Nocturnal hypertension was associated with left ventricular mass index (LVMI) and stroke and silent cerebral infarcts in pediatric renal transplant recipients and children with sickle cell disease, respectively (21, 45). Recently, nocturnal systolic ABP and HBP were shown to be similarly correlated with LVMI, CIMT, carotid dispensability coefficient, and carotid-femoral pulse wave velocity (46).

Seeman et al. (18) reported the association between INH and cardiac damage in children with primary and renal hypertension. They showed that children with INH had higher LVMI adjusted for age than normotensive children and similar LVMI to children with isolated daytime hypertension. Nighttime BP load was the most important predictor of LVMI in their study. In the Cardiovascular Comorbidity in Children with Chronic Kidney Disease Study, INH was associated with CIMT (19).

## Treatment

Since high salt intake is suggested to be a mechanism of INH, salt restriction may be beneficial for INH. Salt restriction and diuretics were reported to have reduced nocturnal BP and shifted BP pattern from non-dipping to dipping (47). In view of the potential contribution of the renin–angiotensin–system to INH, angiotensin converting enzyme inhibitor and or angiotensin receptor blocker (ARB) may provide greater benefit. However, in 411 Japanese patients with nocturnal hypertension, ARB/calcium channel blocker combination was superior to the ARB/diuretic combination independent of sodium intake (48). The MAPEC study compared morning dosing and bedtime dosing. After a follow-up of more than 5 years in 2,156 hypertensive patients, the study reported that the bedtime dosing achieved better BP control (49). No study has examined the treatment target of INH (50). According to the “J curve” theory, clinic BP values should not be below 120 mmHg for systolic BP and below 70 mmHg for diastolic BP. The minimum acceptable value for nocturnal BP is not known. The cutoff values of nocturnal BP that necessitates the initiation of therapy is also unknown.

In pediatric renal transplant recipients with INH, evening dosing of antihypertensive medication reduced nocturnal BP and restored nocturnal BP dip (51).

## Conclusion

Data for INH in children are scarce at present even though ABPM and HBPM are recommended for detecting various conditions including nocturnal hypertension. Recently developed nocturnal BP measurement by HBPM may facilitate the research. Its feasibility has been demonstrated in children. Its reproducibility, reliability, and practicality may prove to be beneficial in clinical practice. Many issues need to be resolved, however, including the establishment of optimal measurement schedules and the reference values for age, gender, and height. The threshold values should be set based on TOD in all age group children. In the long run, therapeutic strategies targeting INH should be established and its effect should be clarified. These are especially important for children with specific diseases including diabetes and CKD who are at higher risk for TOD.

## Author Contributions

MA conceived the idea, performed Medline research, selected the literature, wrote the manuscript, and critically revised it.

## Conflict of Interest

The author declares that the research was conducted in the absence of any commercial or financial relationships that could be construed as a potential conflict of interest.

## Publisher's Note

All claims expressed in this article are solely those of the authors and do not necessarily represent those of their affiliated organizations, or those of the publisher, the editors and the reviewers. Any product that may be evaluated in this article, or claim that may be made by its manufacturer, is not guaranteed or endorsed by the publisher.
